# A six-month weight loss intervention is associated with significant changes in serum biomarkers related to inflammation, bone and cartilage metabolism in obese patients with psoriatic arthritis and matched controls

**DOI:** 10.1186/s41927-025-00511-0

**Published:** 2025-05-23

**Authors:** Linda Torres, Charlotte A. Jonsson, Björn Eliasson, Helena Forsblad-d’Elia, Anton J. Landgren, Annelie Bilberg, Inger Gjertsson, Ingrid Larsson, Eva Klingberg

**Affiliations:** 1 Department of Rheumatology and Inflammation Research, Institute of Medicine, Sahlgrenska Academy, University of Gothenburg, Gothenburg, Sweden; 2 Department of Rheumatology, Sahlgrenska University Hospital, Gröna stråket 16, Gothenburg, 413 45 Sweden; 3 Department of Medicine, Sahlgrenska University Hospital, Gothenburg, Sweden; 4 Region Västra Götaland, Research and Development Primary Health Care, Gothenburg, Södra Bohuslän Sweden; 5 Institute of Neuroscience and Physiology, Department of Health and Rehabilitation, Physiotherapy, Sahlgrenska Academy, University of Gothenburg, Gothenburg, Sweden; 6 Department of Occupational and Physiotherapy, Sahlgrenska University Hospital, Gothenburg, Sweden; 7 Department of Gastroenterology and Hepatology, Sahlgrenska University Hospital, Institute of Medicine, Sahlgrenska Academy, University of Gothenburg, Gothenburg, Sweden

**Keywords:** Biomarkers, Inflammation, Bone remodeling, Psoriatic arthritis, Obesity, Weight loss

## Abstract

**Background:**

Obesity is highly overrepresented in patients with psoriatic arthritis (PsA) and associated with increased disease activity and inferior treatment outcome. We have previously reported in 41 patients with PsA and body mass index (BMI) ≥ 33 kg/m^2^ that weight loss treatment with Very Low Energy Diet (VLED) resulted in a median weight loss of 18,6% and concomitantly a significant improvement in C-reactive protein (CRP) and disease activity at six months (M6). This sub-study analyzes the effects on serum biomarkers associated with inflammation, bone and cartilage metabolism in the same PsA patients and matched controls.

**Methods:**

Patients and controls received VLED treatment (640 kcal/day) during 12–16 weeks depending on baseline (BL) BMI < 40 or ≥ 40 kg/m^2^, followed by an energy restricted diet. Serum was collected at BL and M6, and biomarkers were measured with Magnetic Luminex^®^ Assays and enzyme-linked immunosorbent assay (ELISA). Nonparametric statistics and paired comparison tests were used.

**Results:**

In the PsA patients, the following proteins were significantly reduced at M6 as compared to BL: hepatocyte growth factor (HGF) (median (first-third quartile) 327.9 (250.3-413.6) pg/mL vs. 271.3 (206.9–331.0) pg/mL, *p* < 0.01), vascular endothelial growth factor (VEGF) (79.6 (55.9-113.5) pg/mL vs. 69.6 (53.1-105.3) pg/mL, *p* = 0.01), B-cell activating factor (BAFF) (794.4 (716.4-868.3) pg/mL vs. 674.6 (613.2-790.5) pg/mL, *p* = 0.01) and cartilage oligomeric matrix protein (COMP) (266.1 (209.9–366.0) ng/mL vs. 217.0 (156.0-272.0) ng/mL, *p* < 0.01), whereas carboxyterminal telopeptide of type-1 collagen (CTX-1) was significantly increased (268.0 (196.0-378.5) pg/mL vs. 508.0 (350.0-640.0) pg/mL, *p* < 0.01). Similar results were found in the control group.

**Conclusions:**

Weight loss was associated with reduced levels of serum biomarkers related to inflammation and cartilage degradation, and increased biomarkers for bone resorption. The study supports the strong relationship between obesity, inflammation, bone and cartilage metabolism, identifying BMI as a possible confounder for biomarker levels.

**Trial registration:**

ClinicalTrials.gov identifier: NCT02917434, registered on September 21, 2016, retrospectively registered.

**Supplementary Information:**

The online version contains supplementary material available at 10.1186/s41927-025-00511-0.

## Background

The inflammatory skin disease psoriasis (PsO) affects 2–3% of the population in the western world. Approximately 20–30% of these individuals develop psoriatic arthritis (PsA), a systemic condition manifested by a combination of peripheral arthritis, spondylitis, enthesitis, and dactylitis [1]. A key feature of PsA is pathological bone remodeling affecting not only the mechanism of bone loss but also of bone formation. Nearly half of the patients develop bone erosions in the first two years of disease, and 20% of patients with polyarticular involvement exhibit a severe joint destructive disease [2]. New bone formation causes abnormal calcification in the entheses, spine and peripheral joints.

Obesity is a global health care problem with a strong association to PsA. Excess body weight is an established risk factor for the development of PsO in the general population, and increases the risk of PsA among patients with PsO [3]. A high body mass index (BMI) is linked with increased PsA disease activity and less favorable treatment outcomes, while weight reduction is connected to improvement of the disease [4, 5]. Weight gain and obesity appear to generate a phenotype shift of the white adipose tissue compared to lean individuals, switching the normal well-balanced immune cell homeostasis to a dysfunctional state of chronic low-grade inflammation. Activated macrophages accumulate in the expanding adipose tissue, acting as key mediators for the recruitment of other immune cells and contributing to both a local and a systemic inflammatory response [6, 7]. Traditionally, obesity has been viewed as beneficial for bone structure strength, mainly through the mechanism of mechanical loading. More recent studies have reported negative effects from excessive fat mass on bone mineral density (BMD) and negative influence of systemic inflammation on markers of bone remodeling [8]. The interaction between obesity and bone metabolism is not fully understood [9], and the impact of weight loss on bone in obese PsA patients has not been previously studied.

Further research is needed to understand the complex pathogenesis of PsA and biomarkers have been identified as a relevant research gap [10]. We have already demonstrated that weight reduction with Very Low Energy Diet (VLED) improved parameters of disease activity in patients with PsA and obesity [11]. The same study provided the opportunity to explore the effects on biomarkers related to inflammatory and immunologic processes, bone and cartilage metabolism associated with rheumatologic diseases in previous studies.

The aim of this sub-study was to investigate how weight loss treatment with VLED corresponds with levels of serum biomarkers associated with inflammatory processes, bone and cartilage remodeling in patients with PsA compared to matched controls. The objective was to provide new insights into the interplay between obesity, inflammation, bone and cartilage remodeling in PsA.

## Methods

This is an open prospective interventional study conducted at the Department of Rheumatology and the Regional Obesity Center at Sahlgrenska University hospital in Gothenburg in Western Sweden.

### Patients and controls

Patients with PsA and obesity registered at the Rheumatology clinic of Sahlgrenska University hospital and the rheumatology units at the hospitals of Alingsås and Borås were invited to participate. Eligible for inclusion were patients with PsA fulfilling the Classification for Psoriatic Arthritis (CASPAR) criteria [12], with a BMI ≥ 33 kg/m^2^ and 25–75 years of age. Exclusion criteria were pregnancy, porphyria, epilepsy, type 1 diabetes, severe heart, kidney or catabolic disease, severe binge eating disorder, current treatment with warfarin, lithium or phenytoin, mental imbalance affecting participation, heart infarction, stroke, major surgery or trauma during last the three months, or cancer treatment during the last five years. Background treatment with conventional synthetic and/or biologic disease-modifying anti-rheumatic drugs (cs and/or bDMARDs) had to be constant from three months prior to baseline (BL) until six months after baseline (M6). Recruited as controls were 39 patients with obesity, matched for sex, age and body weight to the PsA patients, already planned for VLED treatment at the Regional Obesity Center at Sahlgrenska University Hospital. In addition to exclusion criteria already accounted for, the controls were excluded if having PsO, PsA or any other inflammatory rheumatic disease. All participants in the study gave their written informed consent. The study was approved by the Regional Ethics Committee in Gothenburg (approval number 901 − 15) and carried out in accordance with the Helsinki Declaration. The trial was registered in ClinicalTrials.gov identifier: NCT02917434.

### The intervention

A structured weight loss treatment was provided within a 12-month protocol including support and medical follow-up from a team of doctors, nurses and dietitians at the Regional Obesity Centre of the Region Western Sweden at Sahlgrenska University Hospital, as described before [11]. The VLED used in the present study provided a daily intake of 640 kcal including recommended doses of vitamins, minerals and other essential nutrients (Cambridge Weight Plan Limited, Corby, UK). All participants followed an initial period of 12 or 16 weeks on strict VLED, based on pre-treatment BMI (< 40 or ≥ 40 kg/m^2^). Following the VLED period, food was gradually reintroduced during a period of 12 weeks, during which each participant received personal dietary advice on a healthy, energy-restricted diet. All participants were seen by a physiotherapist at BL and after six and twelve months and were promoted to engage in health enhancing physical activity during at least 150 min per week.

### Physical assessment

Study visits for PsA patients and controls were carried out at the Clinical Rheumatologic Research Unit at Sahlgrenska University Hospital at BL, after three months and at M6. BMI was calculated. Patients with PsA had the joints evaluated with 66/68 swollen/tender joints count and the entheses according to Leeds enthesitis index [13]. PsA disease activity was assessed with visual analogue scale (VAS) for global disease activity. Function and activity limitations were determined using the Health Assessment Questionnaire (HAQ) [14]. Disease Activity Score using 28 joint counts based on C-reactive protein (DAS28CRP) and the Disease Activity in PSoriatic Arthritis (DAPSA) score were calculated [15, 16]. Minimal disease activity (MDA) was defined as fulfilling five of seven pre-specified criteria [17].

### Biomarkers

**Hepatocyte growth factor** (HGF) is a multi-functional cytokine generating a variety of inflammatory and immune responses, including cytokine production, antigen presentation and T cell effector function [18].

**Vascular endothelial factor** (VEGF) is a potent signaling protein involved in angiogenesis, an important mechanism of chronic inflammatory disorders. VEGF also plays critical roles in skeletal development, bone repair and regeneration [19].

**Matrix metalloproteinases** (MMPs) comprise a family of zinc-dependent enzymes that participate in numerous biological processes such as bone remodeling and several aspects of immunity. MMPs also have an important role in pathological processes such as chronic inflammation and tissue destruction [20].

**B-cell activating factor** (BAFF) participates in B-lymphocyte survival and B- and T-cell maturation [21]. Although PsO and PsA are considered T cell driven diseases, the complex immunologic interplay with B cells have not been defined.

**S100A8 and S100A9** compose a heterocomplex also known as calprotectin. Secreted by activated phagocytes such as neutrophilic granulocytes and monocytes, calprotectin acts as a promotor of the inflammatory response [22].

**Osteocalcin** constitutes the most abundant non-collagenous component of bone. Besides serving as a marker for bone disorders, osteocalcin has also been widely investigated for its regulatory functions on energy metabolism [23].

**Soluble receptor activator of nuclear factor-kB ligand** (RANKL) is a cytokine exhibiting multiple effects on bone metabolism and the immune system. The ratio of RANKL and decoy receptor **osteprotegerin** (OPG) is considered a major determinant for bone mass turnover [24].

**Dickkopf-1** (DKK-1) is proposed to be a master regulator of the osteoblast-osteoclast axis by inhibiting of the Wnt signaling pathway. Vast evidence imply that elevated Dkk-1 expression contributes to the pathogenesis of erosive bone disorders such as PsA [25].

**Sclerostin** (SOST) inhibits bone formation mediated by the Wnt-signaling pathway and have an important role in the adaptive response to mechanical loading in bone [26].

**Cartilage oligomeric matrix protein** (COMP) is an extracellular matrix glycoprotein expressed primarily in cartilage tissue and its release pattern reflects cartilage turnover [27].

**Carboxyterminal telopeptide of type-1 collagen** (CTX-1) are peptide fragments generated by collagen degradation, serving as important biochemical markers of bone resorption [28].

### Laboratory assessment

Sera were obtained from the participants in the morning after ≥ 8 h of fasting and stored at -80 °C. CRP was analyzed using standard laboratory techniques at Sahlgrenska University Hospital. Serum (two-fold dilution) levels were analyzed for HGF, VEGF, S100A8, S100A9, MMP-3, MMP-8, BAFF, Dkk-1, SOST, RANKL, and OPG with Human Magnetic Luminex^®^ Assays (R&D Systems). The Luminex^®^ assays were analyzed using a Bio-Plex 200 system (BioRad) with five-parameter logistic standard curves. Enzyme-linked immunosorbent assay (ELISA) was used to measure serum COMP (Human COMP Quantikine ELISA kit, R&D Systems, serum diluted 1:100), CTX-1 (Serum Crosslaps^®^ (CTX-1) ELISA, Immunodiagnostics systems (IDS), serum undiluted), and osteocalcin (N-MID Osteocalcin ELISA, IDS, serum undiluted). The ELISA plates were analyzed using a Spectramax 340PC384 Microplate Reader (Molecular Devices), and the program SoftMax Pro 5.4.1 with a 4-parameter curve fit was used. All assays were run according to the instructions of the manufacturers.

### Statistical analyses

Descriptive statistics are presented as numbers (%), median and first-third quartile (Q_1_-Q_3_). For comparisons between groups the Mann-Whitney U-test was used for continuous variables and Pearson chi-square test for categorical variables. Wilcoxon signed rank test was used to compare continuous related samples. Correlations were calculated using Spearman’s correlation (r_s_). All tests were two-tailed and *p* ≤ 0.05 was considered statistically significant. Statistical analyses were made using SPSS Statistics version 28.0.1.0 and 29.0.0.0 (IBM, Chicago, USA).

## Results

### Patients and controls characteristics at baseline

Forty-six patients were included in the study. Three patients cancelled participation before follow-up at M6, one patient was excluded due to depression, and one due to pregnancy. A total number of 41 patients and 39 controls completed the intervention and the six-month evaluation. The patient group had a median BL age of 54 (Q_1_-Q_3_ 49–62) years, and 63% were women. The control group consisted of 39 individuals with a median age of 55 (Q_1_-Q_3_ 46–60) years, and 72% were women. They were matched with the patients for sex, age and body weight. A comparison of BL characteristics of the PsA patients and controls is shown in Table 1.

**Table 1:** Characteristics of patients with psoriatic arthritis and controls at baseline

	Patients *N* = 41	Controls *N* = 39	*p*-value
Women, n (%)	26 (63)	28 (72)	0.42
Age, years	54 (49–62)	55 (46–60)	0.46
Body height, cm	168 (162–177)	166 (162–172)	0.19
Body weight, kg	106 (96–114)	105 (97–120)	0.69
BMI, kg/m^2^	35 (34–38)	38 (37–42)	**< 0.01**
Waist circumference, cm	116 (112–122)	117 (107–126)	0.82
Current smoking, n (%)	1 (2)	2 (5)	0.53
PsO duration, years	32 (19–40)		
PsA duration, years	17 (11–27)		
PsA peripheral disease	35 (85)		
SJC 66, score	0 (0–1)		
TJC 68, score	4 (1–14)		
VAS pain, mm	30 (19–63)		
MDA, n (%)	12 (29.3)		
DAPSA, score	15.3 (6.6–29.1)		
DAS28CRP, score	2.9 (2.1–3.7)		
NSAID, n (%)	26 (63)		
csDMARD without biologic, n (%)	17 (41)		
Methotrexate, n (%)	24 (59)		
Sulfasalazine, n (%)	4 (10)		
Leflunomide, n (%)	1 (2)		
Azathioprine, n (%)	1 (2)		
Prednisone, n (%)	3 (7)		
TNFi all, n (%)	13 (32)		
TNFi monotherapy, n (%)	3 (7)		
TNFi + csDMARD, n (%)	10 (24)		
Ustekinumab monotherapy, n (%)	1 (2)		
Apremilast, n (%)	1 (2)		

Bold value in table 1 is to highlight a significant difference in BMI between patients and controls

Figures are number (%) or median and first quartile to third quartile (Q_1_-Q_3_). Values in bold are statistically significant (*p* ≤ 0.05). BMI: body mass index; PsO: psoriasis; PsA: psoriatic arthritis; SJC: swollen joint count; TJC: tender joint count; VAS: visual analogue scale; MDA: minimal disease activity; DAPSA: disease activity in psoriatic arthritis; DAS28CRP: disease activity score using 28 joint counts based on c-reactive protein; NSAID: non-steroidal anti-inflammatory drug; csDMARD: conventional synthetic disease modifying anti-rheumatic drug; TNFi: tumor necrosis factor inhibitor.

### Baseline serum biomarker levels and measures of disease activity

At BL, BAFF correlated positively with tender joint count 68 (r_s_=0.44,*p* < 0.01), DAS28CRP (r_s_=0.54,*p* < 0.01), HAQ (r_s_=0.45,*p* < 0.01), Leeds enthesitis score (r_s_=0.36,*p* = 0.02) and DAPSA score (r_s_=0.49,*p* < 0.01). RANKL correlated positively with number of tender joints 68 (r_s_=0.39,*p* = 0.01) and DAPSA score (r_s_=0.34,*p* = 0.04). Twelve patients (29%) fulfilled criteria for MDA at BL (Table 1). BAFF was the only biomarker associated with MDA at BL (683.3(603.3-788.3)pg/mL(*n* = 12) vs. 815.4(732.0-919.0)pg/mL(*n* = 29),*p* = 0.01).

### Serum levels of biomarkers at month 6 in patients and controls

Six months after the initiation of VLED treatment, the median weight loss for the 41 PsA patients was 18.9 (Q_1_-Q_3_ 15.0-26.5)kg or 19% [15–26], corresponding with the weight reduction achieved in the control group, 22.6 (Q_1_-Q_3_ 14.8–28.5)kg or 20% [14–27]. At M6 significant decreases as compared to BL were found for the PsA patients in serum HGF (*p* < 0.01), BAFF (*p* < 0.01), together with VEGF, S100A8, MMP-8, COMP and Dkk-1, while there was an increase in CTX-1 and SOST (Table 2). The weight loss in the control group was similarly associated with statistically significant reductions in serum HGF, VEGF, BAFF, S100A8, MMP-3, COMP, Dkk-1, and an increase in CTX-1 and SOST (Table 2). At M6, 54% of the PsA patients had reached MDA. SOST was the single biomarker associated with MDA at M6 (62.3(36.6–65.9)pg/mL(*n* = 19) vs. (58.7(36.1–88.9)pg/mL(*n* = 22)*p* = 0.02).

**Table 2:** Comparison of patients with psoriatic arthritis and controls at baseline and 6-month follow-up

	Patients *N* = 41			Controls *N* = 39			Patients vs. controls *p*-value	
	BL	M6	*p*-value	BL	M6	*p*-value	BL	M6
BMI (kg/m^2^)	35.2 (34.1–38.1)	29.8 (26.6–31.5)	**< 0.01**	37.7 (36.7–41.5)	30.4 (27.9–33.2)	**< 0.01**	**< 0.01**	0.11
CRP (mg/L)	4.0 (2.0-8.5)	3.00 (1.5–6.5)	**0.04**	4.00 (2.0–6.0)	2.00 (1.0–4.0)	**< 0.01**	0.28	**0.05**
HGF (pg/mL)	327.9 (250.3-413.6)	271.3 (206.9–331.0)	**< 0.01**	307.9 (239.1-348.2)	239.8 (200.3–276.0)	**< 0.01**	0.40	0.10
VEGF (pg/mL)	79.6 (55.9-113.5)	69.6 (53.1-105.3)	**0.01**	82.3 (48.0-125.9)	65.0 (42.2–85.5)	**< 0.01**	0.96	0.31
S100A8 (pg/mL)	75.5 (48.0-99.5)	63.3 (42.8–93.6)	**0.02**	71.8 (40.5–101.0)	63.3 (40.3–85.7)	**< 0.01**	0.60	0.62
S100A9 (pg/mL)	388.7 (249.2-507.6)	348.8 (249.1-515.7)	0.37	335.3 (262.5-435.1)	308.0 (214.6-394.7)	0.37	0.26	0.18
MMP-3 (µg/mL)	14.3 (9.5–22.0)	12.48 (8.2–19.7)	0.42	13.2 (10.0-18.5)	12.3 (9.3–15.8)	**0.02**	0.73	0.60
MMP-8 (ng/mL)	10.0 (6.8–14.2)	9.2 (5.8–12.0)	**0.02**	7.5 (4.8–12.6)	7.2 (3.5–9.8)	0.11	0.051	**0.047**
BAFF (pg/mL)	794.4 (716.4-868.3)	674.6 (613.2-790.5)	**< 0.01**	760.8 (664.1-827.3)	678.1 (603.7-719.8)	**< 0.01**	0.17	0.49
COMP (ng/mL)	266.1 (209.9–366.0)	217.0 (156.0-272.0)	**0.01**	293.6 (185.2-340.5)	221.6 (163.5–300.0)	**0.02**	0.95	0.73
Dkk-1 (ng/mL)	3.6 (3.1–4.4)	3.4 (2.8–4.2)	**< 0.01**	3.6 (3.2–4.4)	3.5 (2.9–4.1)	**< 0.01**	0.88	0.93
SOST (pg/mL)	52.9 (32.5–65.4)	60.3 (37.2–85.6)	**0.01**	50.0 (30.8–79.3)	61.3 (35.7–81.4)	**0.02**	0.92	0.91
CTX-1 (pg/mL)	268.0 (196.0-378.5)	508.0 (350.0-644.0)	**< 0.01**	226.0 (160.0-338.0)	499.0 (301.0-610.0)	**< 0.01**	0.12	0.30
OPG (pg/mL)	797.0 (682.1–1037.0)	822.8 (680.1–966.0)	0.23	822.9 (699.0-948.1)	754.1 (643.0-904.3)	0.061	0.62	0.24
RANKL (pg/mL)	31.3 (17.4–47.1)	29.42 (18.6–44.8)	0.75	28.8 (23.6–44.1)	29.9 (19.8–36.0)	**0.02**	0.76	0.70
Osteocalcin	12.51 (9.68–16.92)	15.46 (10.63–18.71)	0.06	10.65 (8.23–15.46)	12.80 (9.24–17.82)	0.07	0.10	0.22

Figures are median and first to third quartile (Q_1_-Q_3_). Values in bold are statistically significant (*p* ≤ 0.05). BMI: body mass index; CRP: c-reactive protein; HGF: hepatocyte growth factor; VEGF: vascular endothelial growth factor; MMP-3: matrix metalloproteinase 3; MMP-8: matrix metalloproteinase 8; BAFF B-cell activating factor; COMP: cartilage oligomeric matrix protein; Dkk-1: dickkopf-1; SOST: sclerostin; CTX-1: carboxy-terminal crosslinked telopeptide of type 1 collagen; OPG: osteoprotegerin; RANKL: receptor activator of nuclear factor kappa B ligand

**Fig. 1:**
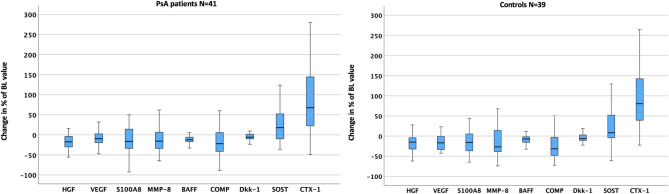
Significant change (%) in serum levels of biomarkers from baseline to month six

**Fig. 2:**
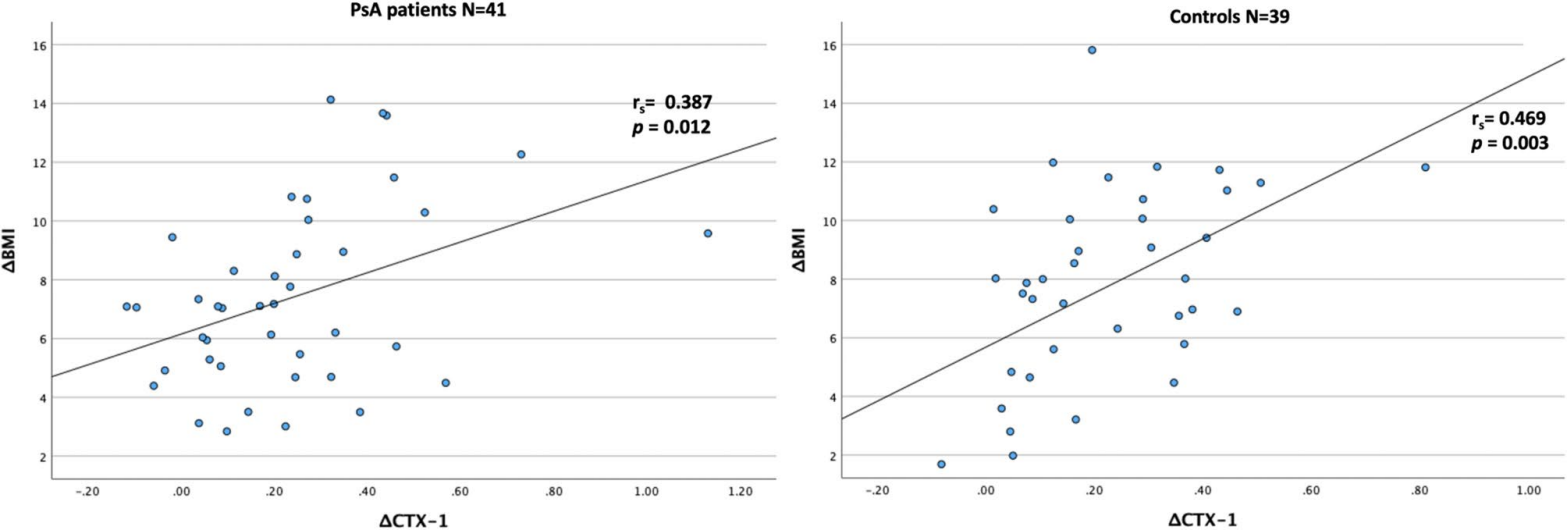
Change in CTX-1 between baseline and month six in relation to delta body mass index

Figure 1 illustrates the biomarkers with significant delta values from BL to M6 found in the PsA patients, compared with the controls. Figure 2 highlights the changes in CTX-1 serum levels in relation to delta BMI (∆BMI) in both groups.

### Change in serum levels of biomarker in relation to PsA disease activity at month 6

The reduction in BMI correlated positively with ∆CRP. The decrease of MMP-8 correlated with a significant improvement of swollen joint counts 66. The bone resorption biomarker ∆CTX-1 had an inverse correlation with the ∆values of BMI, swollen joint count 66 and DAS28CRP activity score. ∆RANKL was positively associated with improvement in ∆DAS28CRP score. Table 3 depicts the ∆values of biomarkers with at least one significant correlation to the ∆values of PsA disease activity parameters. The magnitude of ∆values of the biomarkers were similar in patients and controls.

**Table 3:** Changes in biomarkers, body mass index and disease activity in 41 patients with psoriatic arthritis

		∆BMI (kg/m^2^)	∆Swollen joint count 66 (score)	∆Tender joint count 68 (score)	∆DAS28CRP (score)	∆BSA (score)	DAPSA (score)
∆CRP (mg/L)	r_s_ *p*-value	0.34 **0.03**	0.02 0.89	-0.20 0.22	0.13 0.42	0.14 0.39	-0.11 0.48
∆MMP-8 (ng/mL)	r_s_ *p*-value	0.15 0.35	0.39 **0.01**	0.07 0.64	0.07 0.68	0.06 0.72	0.10 0.52
∆CTX1 (pg/mL)	r_s_ p-value	-0.39 **0.01**	-0.35 **0.03**	-0.06 0.71	-0.45 **< 0.01**	-0.30 0.06	-0.19 0.25
∆RANKL (pg/mL)	r_s_ *p*-value	0.31 0.06	-0.06 0.72	0.32 0.05	0.35 **0.03**	0.13 0.42	0.30 0.06

Values in bold are statistically significant (*p* ≤ 0.05). CRP: c-reactive protein; MMP-8: matrix metalloproteinase 8; CTX-1: carboxy-terminal crosslinked telopeptide of type 1 collagen; RANKL: receptor activator of nuclear factor kappa B ligand; BMI: body mass index; DAS28CRP: disease activity score using 28 joint counts based on c-reactive protein; BSA: body surface area; DAPSA: disease activity in psoriatic arthritis

## Discussion

In this prospective, open intervention study we analyzed the effects of weight loss with a VLED regimen on serum biomarkers reflecting inflammation, bone and cartilage metabolism in patients with PsA and obesity and matched controls. All biomarkers were included in each of the analyzes. Non-significant findings are not always described in the results. Discrepancy in BMI at BL is explained by an otherwise non-significant difference in body height. At M6 the patients displayed significant reductions of serum biomarkers CRP, HGF, VEGF, MMP-8, BAFF, S100A8, COMP and Dkk-1, whereas serum SOST and CTX-1 were increased. Similar changes in biomarker levels were seen in the control group. Irrespective of the group, weight loss led to a reduction in pro-inflammatory molecules, a decrease in biomarkers of cartilage degradation and an increase in biomarkers reflecting bone resorption.

The results showed that weight loss treatment was associated with decreases in serum HGF in both PsA patients and controls. Previous research on patients with rheumatoid arthritis (RA) have demonstrated elevated HGF levels in serum, synovium, synovial fluid and joint adipose tissue, and that above-median levels of plasma HGF predicted progress in radiographic joint damage [29, 30]. HGF serum levels were significantly reduced after weight reduction through bariatric surgery [31]. The lowering of serum HGF in the current study could likely be primarily related to a depletion of adipose tissue, and may also reflect an alleviation of mechanical stress on the joints and reduced inflammatory activity.

The weight loss was accompanied by a decrease in serum VEGF in both PsA patients and controls. Ramonda et al. described significantly higher serum VEGF levels in PsA patients compared to matched healthy controls [32]. Elevated VEGF levels have been observed in obese men and women, highly correlating with BMI [33].

In the present study MMP-8 was significantly reduced in the PsA patients and ΔMMP-8 levels correlated with improvement of swollen joint count. Mattey et al. reported that MMP-8 had a strong association with disease activity and worse function in ankylosing spondylitis (AS) [34]. Otherwise, there are few previous studies on MMP-8 and arthritis. Bariatric surgery has been associated with a significant reduction of MMP-8 levels, correlating with lowered serum leptin [35].

VLED treatment was associated with a significant reduction of BAFF in both the PsA patients and the control group. We found correlations between serum BAFF and several disease activity parameters at BL. However, following weight loss, ΔBAFF did not correlate with change in disease activity variables. Previous studies on PsA and PsO patients have shown that interleukin (IL)-10-producing regulatory B cells (B_regs_) were decreased and inversely correlated with T helper (T_h_) 17 and Th 1 cells compared with healthy controls [36]. Conigliaro et al. reported improvement of joint activity along with increased levels of peripheral blood B cells from TNFi treatment in both RA and PsA patients [37]. BAFF has been proposed to function as a regulator of adipokines and a possible mediator between adipocytes and macrophages [38]. Further studies on BAFF in relation to obesity and weight loss in other rheumatic diseases such as SLE and RA could be of interest.

In our study, weight reduction resulted in a significant decrease of S100A8 in both the PsA patient and the control group. There was no correlation with S100A8/S100A9 and PsA disease parameters. Previously, conflicting results have been shown regarding serum calprotectin levels and PsA disease activity. In a study on 1729 subjects with RA, axial spondyloarthritis (axSpA) and PsA by Jarlborg et al., serum calprotectin levels correlated positively with disease activity in RA and axSpA, but not in PsA [39]. In contrast, a recent Italian study demonstrated a correlation between serum calprotectin and ultrasound-detected synovitis in early PsA [40].

Sclerostin increased after weight loss treatment in both the patient and the control group. In line with our results, a study by Muschitz et al. have demonstrated an increase in sclerostin and CTX1 in obese women after bariatric surgery [41]. Research have indicated that patients with AS exhibit an impaired SOST expression, and a low serum level has been linked to persistent inflammation and formation of new syndesmophytes [42]. The outcome could indicate bone loss due to the adaptive response to lessened mechanical loading but may also be an effect of reduced pathological bone remodeling.

Dkk-1 was reduced in response to weight loss treatment in both the PsA patient and control group. While our study found no significant difference in serum levels of Dkk-1 between the patient and the control group, previous studies have reported inconsistent results [43, 44]. Wnt signaling has been suggested to have fundamental roles in the development of adipose tissue by influencing the differentiation of mesenchymal stem cells [45]. A previous study also proposed that Dkk-1 is secreted by human preadipocytes and functions as a promoter of adipogenesis. Inhibition of Wnt signaling promotes adipogenic differentiation [46]. Hence, our findings could be associated with the reduction of adipose tissue.

Previous studies on COMP and PsA are inconclusive [47, 48]. Studies on osteoarthritis have acknowledged a sensitivity of COMP to mechanical loading [49]. The significant decrease of serum COMP in both patients and controls could most likely be related to the reduction of mechanical stress on cartilage.

Grisar et al. reported higher CTX-1 levels in PsA patients compared to controls, and significant correlations between CTX-1 and inflammatory markers, as well as PsA disease duration [50]. In our study, CTX-1 levels increased in response to weight loss in both PsA patients and the control group. ∆CTX1 correlated with a significant improvement of swollen joint count 66 and DAS28CRP score. The findings suggest an increase in bone resorption following weight loss therapy, in agreement with the results from the previously mentioned study, reporting increased CTX-1 levels after bariatric surgery [41]. Long-term studies on CTX-1 after weight loss and its relation to bone loss and increased fracture risk could be of interest.

### Limitations and strengths

The findings in this study should be interpreted in light of some limitations. This is not a randomized trial. However, a control group of individuals without PsA, matched for age, sex and weight, subjected to the same VLED treatment was included in the study. Treatment with cs/bDMARD could be a confounding factor. Patients exhibiting a more severe disease receive more intense treatment, which possibly could affect the biomarkers. Low disease activity could taper the effects of the weight reduction on symptoms of disease activity in the patient group. The Leeds enthesitis score is a simple and common method to assess enthesitis, but is limited by few examination points and the inability to distinguish between tenderness and inflammation. There was a difference in BMI at BL due to an otherwise non-significant difference in body height between the patient and control group. The effects on serum biomarkers and disease activity variables could be altered by the state of temporary starvation. Also, biomarkers S100A8, RANKL and SOST were expressed at very low levels, and serum levels determined from an extrapolated part of the standard curve. Due to the exploratory nature of this study, the authors have decided not to adjust for multiplicity to limit the risk for type II errors, which must be considered when interpretating the results.

Strengths of the present study include its prospective design, effective intervention method, structured regime of follow-up, low attrition rate and excellent adherence to the dietary treatment leading to a substantial weight loss. Another important strength is the generalizability of the study, including a wide range of every-day care PsA patients. To minimize impact of treatment change on the biomarkers, cs/bDMARDs were held unchanged from three months prior to BL until M6. Blood samples were collected fasting, and early in the morning in account for the circadian rhythm. BL and M6 samples from each participant were run together in the same plate to limit analytical variation. Patients were examined by the same physician for a consistent estimation of joint activity.

## Conclusions

A structured weight loss treatment in patients with PsA and obesity was accompanied by significant changes in several biomarkers associated with inflammation, bone and cartilage metabolism. Serum levels of HGF, VEGF, BAFF and COMP had a particular significant decrease in response to the weight loss, while CTX-1 was significantly increased. In the PsA patients, ∆CTX-1 correlated with ∆BMI and ∆swollen joint 66 count and ∆DAS28CRP. Similar changes in the biomarkers were observed in the control group, and a comparative analysis displayed no difference between the PsA patient and control group. Hence, the results of this sub-study suggest that the beneficial effect of weight loss treatment through VLED on PsA disease activity parameters in the previous report [11], might be related to factors associated with obesity, rather than with the PsA disease per se.

This study supports the hypothesis of obesity as a promotor of inflammation, cartilage and bone remodeling, with association to parameters of disease activity in PsA. The results also bring attention to the need to consider adjusting for BMI in the evaluation of serological biomarkers.

## Electronic supplementary material

Below is the link to the electronic supplementary material.

Supplementary Material 1

## Data Availability

The datasets analyzed during the current study are available from the corresponding author on reasonable request.

## Abbreviations

AS: Ankylosing Spondylitis
axSpA: Axial Spondyloarthritis
BAFF: B-cell Activating Factor
BL: Baseline
BMC: Bone Mineral Content
BMD: Bone Mineral Density
BMI: Body Mass Index
B_reg_: Regulatory B Cell
BSA: Body Surface Area
CASPAR: Classification for Psoriatic Arthritis
COMP: Cartilage Oligomeric Matrix Protein
CRP: C-Reactive Protein
CTX-1: Carboxy-terminal crosslinked telopeptide of type 1 collagen
cs/b DMARD: Conventional synthetic/biologic Disease Modifying Anti- Rheumatic Drug
DAS28CRP: Disease Activity Score using 28 joint counts based on CRP
DAPSA: Disease Activity in PSoriatic Arthritis
Dkk-1: Dickkopf-1
HAQ: Health Assessment Questionnaire
ELISA: Enzyme-Linked Immunosorbent Assay
HGF: Hepatocyte Growth Factor
IL-10: Interleukin-10
M6: Month 6
MDA: Minimal Disease Activity
MMP: Matrix Metalloproteinase
NSAID: Non-Steroidal Anti-Inflammatory Drug
OPG: Osteoprotegerin
PsA: Psoriatic Arthritis
PsO: Psoriasis
r_s_: Spearman´s correlation
RA: Rheumatoid Arthritis
RANKL: Soluble Receptor Activator of Nuclear Factor-kB Ligand
SOST: Sclerostin
SJC: Swollen Joint Count
T_h_: T helper Cells
TJC: Tender Joint Count
TNFi: Tumor Necrosis Factor inhibitor
OPG: Osteoprotegerin
VAS: Visual Analogue Scale
VEGF: Vascular Endothelial Growth Factor
VLED: Very Low Energy Diet

## References

[1] Gladman DD, Antoni C, Mease P, Clegg DO, Nash P. Psoriatic arthritis: epidemiology, clinical features, course, and outcome. Ann Rheum Dis. 2005;64(Suppl 2):ii14–7. 15708927 10.1136/ard.2004.032482 PMC1766874

[2] Mc Ardle A, Flatley B, Pennington SR, FitzGerald O. Early biomarkers of joint damage in rheumatoid and psoriatic arthritis. Arthritis Res Ther. 2015;17:141. 26028339 10.1186/s13075-015-0652-z PMC4450469

[3] Husni ME. Comorbidities in psoriatic arthritis. Rheum Dis Clin North Am. 2015;41(4):677–98. 26476226 10.1016/j.rdc.2015.07.008

[4] Kumthekar A, Ogdie A. Obesity and psoriatic arthritis: A narrative review. Rheumatol Ther. 2020;7(3):447–56. 32495313 10.1007/s40744-020-00215-6 PMC7410935

[5] Klingberg E, Bjorkman S, Eliasson B, Larsson I, Bilberg A. Weight loss is associated with sustained improvement of disease activity and cardiovascular risk factors in patients with psoriatic arthritis and obesity: a prospective intervention study with two years of follow-up. Arthritis Res Ther. 2020;22(1):254. 33092646 10.1186/s13075-020-02350-5 PMC7583178

[6] Anandarajah AP, Schwarz EM, Totterman S, Monu J, Feng CY, Shao T, et al. The effect of etanercept on osteoclast precursor frequency and enhancing bone marrow oedema in patients with psoriatic arthritis. Ann Rheum Dis. 2008;67(3):296–301. 17967829 10.1136/ard.2007.076091

[7] Hachiya R, Tanaka M, Itoh M, Suganami T. Molecular mechanism of crosstalk between immune and metabolic systems in metabolic syndrome. Inflamm Regen. 2022;42(1):13. 35490239 10.1186/s41232-022-00198-7 PMC9057063

[8] Cao JJ. Effects of obesity on bone metabolism. J Orthop Surg Res. 2011;6:30. 21676245 10.1186/1749-799X-6-30 PMC3141563

[9] Paine A, Ritchlin C. Altered bone remodeling in psoriatic disease: new insights and future directions. Calcif Tissue Int. 2018;102(5):559–74. 29330560 10.1007/s00223-017-0380-2 PMC5906143

[10] Ryan C, Korman NJ, Gelfand JM, Lim HW, Elmets CA, Feldman SR, et al. Research gaps in psoriasis: opportunities for future studies. J Am Acad Dermatol. 2014;70(1):146–67. 24126079 10.1016/j.jaad.2013.08.042

[11] Klingberg E, Bilberg A, Bjorkman S, Hedberg M, Jacobsson L, Forsblad-d’Elia H, et al. Weight loss improves disease activity in patients with psoriatic arthritis and obesity: an interventional study. Arthritis Res Ther. 2019;21(1):17. 30635024 10.1186/s13075-019-1810-5 PMC6330463

[12] Taylor W, Gladman D, Helliwell P, Marchesoni A, Mease P, Mielants H, et al. Classification criteria for psoriatic arthritis: development of new criteria from a large international study. Arthritis Rheum. 2006;54(8):2665–73. 16871531 10.1002/art.21972

[13] Healy PJ, Helliwell PS. Measuring clinical enthesitis in psoriatic arthritis: assessment of existing measures and development of an instrument specific to psoriatic arthritis. Arthritis Rheum. 2008;59(5):686–91. 18438903 10.1002/art.23568

[14] Fries JF, Spitz P, Kraines RG, Holman HR. Measurement of patient outcome in arthritis. Arthritis Rheum. 1980;23(2):137–45. 7362664 10.1002/art.1780230202

[15] Prevoo ML, van ‘t Hof MA, Kuper HH, van Leeuwen MA, van de Putte LB, van Riel PL. Modified disease activity scores that include twenty-eight-joint counts. Development and validation in a prospective longitudinal study of patients with rheumatoid arthritis. Arthritis Rheum. 1995;38(1):44–8. 7818570 10.1002/art.1780380107

[16] Schoels M, Aletaha D, Funovits J, Kavanaugh A, Baker D, Smolen JS. Application of the DAREA/DAPSA score for assessment of disease activity in psoriatic arthritis. Ann Rheum Dis. 2010;69(8):1441–7. 20525844 10.1136/ard.2009.122259

[17] Coates LC, Fransen J, Helliwell PS. Defining minimal disease activity in psoriatic arthritis: a proposed objective target for treatment. Ann Rheum Dis. 2010;69(1):48–53. 19147615 10.1136/ard.2008.102053

[18] Molnarfi N, Benkhoucha M, Funakoshi H, Nakamura T, Lalive PH. Hepatocyte growth factor: A regulator of inflammation and autoimmunity. Autoimmun Rev. 2015;14(4):293–303. 25476732 10.1016/j.autrev.2014.11.013

[19] Melincovici CS, Bosca AB, Susman S, Marginean M, Mihu C, Istrate M, et al. Vascular endothelial growth factor (VEGF) - key factor in normal and pathological angiogenesis. Rom J Morphol Embryol. 2018;59(2):455–67. 30173249

[20] Nagase H, Woessner JF. Jr. Matrix metalloproteinases. J Biol Chem. 1999;274(31):21491–4. 10419448 10.1074/jbc.274.31.21491

[21] Ng LG, Sutherland AP, Newton R, Qian F, Cachero TG, Scott ML, et al. B cell-activating factor belonging to the TNF family (BAFF)-R is the principal BAFF receptor facilitating BAFF costimulation of Circulating T and B cells. J Immunol. 2004;173(2):807–17. 15240667 10.4049/jimmunol.173.2.807

[22] Odink K, Cerletti N, Bruggen J, Clerc RG, Tarcsay L, Zwadlo G, et al. Two calcium-binding proteins in infiltrate macrophages of rheumatoid arthritis. Nature. 1987;330(6143):80–2. 3313057 10.1038/330080a0

[23] Nowicki JK, Jakubowska-Pietkiewicz E, Osteocalcin. Beyond Bones Endocrinol Metab (Seoul). 2024;39(3):399–406. 38803289 10.3803/EnM.2023.1895 PMC11220208

[24] Ono T, Hayashi M, Sasaki F, Nakashima T. RANKL biology: bone metabolism, the immune system, and beyond. Inflamm Regen. 2020;40:2. 32047573 10.1186/s41232-019-0111-3 PMC7006158

[25] Jadon DR, Nightingale AL, McHugh NJ, Lindsay MA, Korendowych E, Sengupta R. Serum soluble bone turnover biomarkers in psoriatic arthritis and psoriatic spondyloarthropathy. J Rheumatol. 2015;42(1):21–30. 25362660 10.3899/jrheum.140223

[26] Omran A, Atanasova D, Landgren F, Magnusson P. Sclerostin: from molecule to clinical biomarker. Int J Mol Sci. 2022;23(9). 10.3390/ijms23094751 PMC9104784 35563144

[27] Muller G, Michel A, Altenburg E. COMP (cartilage oligomeric matrix protein) is synthesized in ligament, tendon, meniscus, and articular cartilage. Connect Tissue Res. 1998;39(4):233–44. 11063004 10.3109/03008209809021499

[28] Garnero P, Delmas PD. Biochemical markers of bone turnover. Applications for osteoporosis. Endocrinol Metab Clin North Am. 1998;27(2):303–23. 9669140 10.1016/s0889-8529(05)70007-4

[29] Kontny E, Prochorec-Sobieszek M. Articular adipose tissue resident macrophages in rheumatoid arthritis patients: potential contribution to local abnormalities. Rheumatology (Oxford). 2013;52(12):2158–67. 24014647 10.1093/rheumatology/ket287

[30] Grandaunet B, Syversen SW, Hoff M, Sundan A, Haugeberg G, van Der Heijde D, et al. Association between high plasma levels of hepatocyte growth factor and progression of radiographic damage in the joints of patients with rheumatoid arthritis. Arthritis Rheum. 2011;63(3):662–9. 21360495 10.1002/art.30163

[31] Bell LN, Ward JL, Degawa-Yamauchi M, Bovenkerk JE, Jones R, Cacucci BM, et al. Adipose tissue production of hepatocyte growth factor contributes to elevated serum HGF in obesity. Am J Physiol Endocrinol Metab. 2006;291(4):E843–8. 16757549 10.1152/ajpendo.00174.2006

[32] Ramonda R, Modesti V, Ortolan A, Scanu A, Bassi N, Oliviero F, et al. Serological markers in psoriatic arthritis: promising tools. Exp Biol Med (Maywood). 2013;238(12):1431–6. 24146263 10.1177/1535370213506435

[33] Silha JV, Krsek M, Sucharda P, Murphy LJ. Angiogenic factors are elevated in overweight and obese individuals. Int J Obes (Lond). 2005;29(11):1308–14. 15953938 10.1038/sj.ijo.0802987

[34] Mattey DL, Packham JC, Nixon NB, Coates L, Creamer P, Hailwood S, et al. Association of cytokine and matrix metalloproteinase profiles with disease activity and function in ankylosing spondylitis. Arthritis Res Ther. 2012;14(3):R127. 22640827 10.1186/ar3857 PMC3446508

[35] Liberale L, Bonaventura A, Carbone F, Bertolotto M, Contini P, Scopinaro N, et al. Early reduction of matrix metalloproteinase-8 serum levels is associated with leptin drop and predicts diabetes remission after bariatric surgery. Int J Cardiol. 2017;245:257–62. 28734574 10.1016/j.ijcard.2017.07.044

[36] Mavropoulos A, Varna A, Zafiriou E, Liaskos C, Alexiou I, Roussaki-Schulze A, et al. IL-10 producing Bregs are impaired in psoriatic arthritis and psoriasis and inversely correlate with IL-17- and IFNgamma-producing T cells. Clin Immunol. 2017;184:33–41. 28461105 10.1016/j.clim.2017.04.010

[37] Conigliaro P, Triggianese P, Perricone C, Chimenti MS, Di Muzio G, Ballanti E, et al. Restoration of peripheral blood natural killer and B cell levels in patients affected by rheumatoid and psoriatic arthritis during etanercept treatment. Clin Exp Immunol. 2014;177(1):234–43. 24666401 10.1111/cei.12335 PMC4089172

[38] Kim YH, Choi BH, Cheon HG, Do MS. B cell activation factor (BAFF) is a novel adipokine that links obesity and inflammation. Exp Mol Med. 2009;41(3):208–16. 19293640 10.3858/emm.2009.41.3.024 PMC2679246

[39] Jarlborg M, Courvoisier DS, Lamacchia C, Martinez Prat L, Mahler M, Bentow C, et al. Serum calprotectin: a promising biomarker in rheumatoid arthritis and axial spondyloarthritis. Arthritis Res Ther. 2020;22(1):105. 32375861 10.1186/s13075-020-02190-3 PMC7201559

[40] Sakellariou G, Lombardi G, Vitolo B, Gomarasca M, Faraldi M, Caporali R, et al. Serum calprotectin as a marker of ultrasound-detected synovitis in early psoriatic and rheumatoid arthritis: results from a cross-sectional retrospective study. Clin Exp Rheumatol. 2019;37(3):429–36. 30299248

[41] Muschitz C, Kocijan R, Marterer C, Nia AR, Muschitz GK, Resch H, et al. Sclerostin levels and changes in bone metabolism after bariatric surgery. J Clin Endocrinol Metab. 2015;100(3):891–901. 25490275 10.1210/jc.2014-3367

[42] Appel H, Ruiz-Heiland G, Listing J, Zwerina J, Herrmann M, Mueller R, et al. Altered skeletal expression of sclerostin and its link to radiographic progression in ankylosing spondylitis. Arthritis Rheum. 2009;60(11):3257–62. 19877044 10.1002/art.24888

[43] Dalbeth N, Pool B, Smith T, Callon KE, Lobo M, Taylor WJ, et al. Circulating mediators of bone remodeling in psoriatic arthritis: implications for disordered osteoclastogenesis and bone erosion. Arthritis Res Ther. 2010;12(4):R164. 20796300 10.1186/ar3123 PMC2945067

[44] Chung Y, Li ZC, Sun XL, Liu YY, Shao M, Gan YZ, et al. Elevated serum Dickkopf-1 is a biomarker for bone erosion in patients with psoriatic arthritis. Chin Med J (Engl). 2021;134(21):2583–8. 34267065 10.1097/CM9.0000000000001612 PMC8577657

[45] Prestwich TC, Macdougald OA. Wnt/beta-catenin signaling in adipogenesis and metabolism. Curr Opin Cell Biol. 2007;19(6):612–7. 17997088 10.1016/j.ceb.2007.09.014 PMC2709272

[46] Christodoulides C, Laudes M, Cawthorn WP, Schinner S, Soos M, O’Rahilly S, et al. The Wnt antagonist Dickkopf-1 and its receptors are coordinately regulated during early human adipogenesis. J Cell Sci. 2006;119(Pt 12):2613–20. 16763196 10.1242/jcs.02975 PMC4304001

[47] Skoumal M, Haberhauer G, Fink A, Steiner A, Klingler A, Varga F, et al. Increased serum levels of cartilage oligomeric matrix protein in patients with psoriasis vulgaris: a marker for unknown peripheral joint involvement? Clin Exp Rheumatol. 2008;26(6):1087–90. 19210875

[48] Chandran V, Cook RJ, Edwin J, Shen H, Pellett FJ, Shanmugarajah S, et al. Soluble biomarkers differentiate patients with psoriatic arthritis from those with psoriasis without arthritis. Rheumatology (Oxford). 2010;49(7):1399–405. 20421218 10.1093/rheumatology/keq105

[49] Mundermann A, Dyrby CO, Andriacchi TP, King KB. Serum concentration of cartilage oligomeric matrix protein (COMP) is sensitive to physiological Cyclic loading in healthy adults. Osteoarthritis Cartilage. 2005;13(1):34–8. 15639635 10.1016/j.joca.2004.09.007

[50] Grisar J, Bernecker PM, Aringer M, Redlich K, Sedlak M, Wolozcszuk W, et al. Ankylosing spondylitis, psoriatic arthritis, and reactive arthritis show increased bone resorption, but differ with regard to bone formation. J Rheumatol. 2002;29(7):1430–6. 12136902

