# Supplementary material for: A six-month weight loss intervention is associated with significant changes in serum biomarkers related to inflammation, bone and cartilage metabolism in obese patients with psoriatic arthritis and matched controls
Source: BMC Rheumatol. 2025 May 23;9:58. doi: 10.1186/s41927-025-00511-0 (PMC12100911; doi:10.1186/s41927-025-00511-0)
Supplement: Supplementary file 1 — Supplementary Material 1 [file 41927_2025_511_MOESM1_ESM.docx]

*Supplementary table 1*

| **Biomarker** | **Main function** | | **Test kit** | **Mfr** | **Serum dilution** | **Detection range^1^** | **Samples below LLOQ** | | | | **Mean and acceptable range of QC^2^** |
| --- | --- | --- | --- | --- | --- | --- | --- | --- | --- | --- | --- |
|  |  |  |  |  |  |  | **Patients**  **N=41**  **BL M6** | | **Controls**  **N=39**  **BL M6** | |  |
| **ELISA (ng/mL)** | | | | | | | | | | | |
| CTX-1 | Bone | Bone resorption | Serum Crosslaps®  CTX-1 | IDS | No dilution | 0.020 – 2.276 | N= 0 | N= 0 | N= 0 | N= 0 | QC1 range: 0.213 – 0.319  Plate 1: 0.273 ± 0.008  Plate 2: 0.275± 0.016  QC2 range: 0.799 – 1.199  Plate 1: 0.971 ± 0.003  Plate 2: 0.922 ± 0.04 |
| Osteocalcin | Bone | Bone formation | Osteocalcin |  |  | 0.5-97.5 | N= 0 | N= 0 | N= 0 | N= 0 | QC1 range: 20.5 – 30.8  Plate 1: 23.841±0.576  Plate 2: 23.71 ± 0.475  QC2 range: 40.0 – 60.0  Plate 1: 44.041 ±1.46  Plate 2: 44.287 ± 1.437 |
| COMP | Cartilage | Cartilage matrix protein | Human COMP Quantikine ELISA Kit  DCMP0 | R&D systems/ BioTechne | 1/100 | 0.010 – 0.156 | N= 0 | N= 0 | N= 0 | N= 0 | Not applied  by the mfr |
| **Luminex**® **(pg/mL)** | | | | | | | | | | | |
| VEGF | Inflam. | Angio-genesis | LXSAHM-06 | R&D systems/ BioTechne | 1/2 | 7 – 1 640 | N= 0 | N= 0 | N= 0 | N= 0 | Not applied  by the mfr |
| OPG | Bone | Bone formation |  |  |  | 74-17 940 | N= 0 | N= 0 | N= 0 | N= 0 |  |
| HGF | Inflam. | Multi-functional | LXSAHM-25 |  |  | 15-3 660 | N= 0 | N= 0 | N= 0 | N= 0 |  |
| MMP-3 | Inflam. | Multi-functional |  |  |  | 82-20 000 | N= 0 | N= 0 | N= 0 | N= 0 |  |
| MMP-8 | Inflam. | Multi-functional |  |  |  | 222-54 080 | N= 0 | N= 0 | N= 0 | N= 0 |  |
| BAFF | Inflam. | B-cell survival |  |  |  | 17-4110 | N= 0 | N= 0 | N= 0 | N= 0 |  |
| Dkk-1 | Bone | Bone resorption |  |  |  | 202- 49 060 | N= 0 | N= 0 | N= 0 | N= 0 |  |
| SOST | Bone | Bone resorption |  |  |  | 10-2 290 | N= 1 | N= 1 | N= 1 | N= 1 |  |
| RANKL | Bone | Bone resorption |  |  |  | 48-11 680 | N= 2 | N= 2 | N= 1 | N= 2 |  |
| S100A8 | Inflam. | Neutrophil activation |  |  |  | 16 270 – 66 955 | N= 2 | N= 2 | N= 6 | N= 7 |  |
| S100A9 | Inflam. | Neutrophil activation | LXSAHM-01 |  |  | 5 750 – 23 663 | BL: N= 1 | M6: N= 3 | BL: N= 0 | M6: N= 0 |  |

^1^ according to the manufacturer’s instructions

^2^ included in the kits

Mfr: manufacturer; LLOQ: lower limit of quantification; BL: baseline; M6: month 6; QC: quality control; ELISA: Enzyme-linked immunosorbent assay; CTX-1: carboxy-terminal crosslinked telopeptide of type 1 collagen; IDS: Immunodiagnostics systems; COMP: cartilage oligomeric matrix protein; VEGF: vascular endothelial growth factor; Inflam.: Inflammation; OPG: osteoprotegerin; HGF: hepatocyte growth factor; MMP-3: matrix metalloproteinase 3; MMP-8: matrix metalloproteinase 8; BAFF: B-cell activating factor; Dkk-1: dickkopf-1; SOST: sclerostin; RANKL: receptor activator of nuclear factor kappa B ligand.
